# Virtual community-based interventions for family caregivers of people with dementia: A scoping review protocol

**DOI:** 10.1371/journal.pone.0347798

**Published:** 2026-06-24

**Authors:** Yifan Wang, Lei Zhao, Fangyu Zou, Hangyu Zhou, Xiaoyan Li, Caifu Li, Linyan Xu

**Affiliations:** School of Medicine and Health, Lishui University, Lishui, China; Manipal Academy of Higher Education, INDIA

## Abstract

**Introduction:**

Family caregivers provide the majority of long-term care for people with dementia and often experience substantial psychological distress, social isolation, and caregiving burden. With the rapid development of digital technologies, virtual communities—platforms that enable interaction, information exchange, and emotional support—have emerged as a potential approach to support dementia caregivers. However, evidence regarding technological forms, content components and outcomes remains fragmented and heterogeneous. This scoping review aims to systematically map the existing evidence on virtual community-based interventions for family caregivers of people with dementia.

**Methods:**

This protocol follows the Joanna Briggs Institute methodological framework for scoping reviews and will adhere to the PRISMA-P guidelines. The ‘Population-Concept-Context’ framework will guide eligibility determination. Empirical studies involving family caregivers of people with dementia and describing interventions delivered through interactive virtual communities will be eligible. We will systematically search eight databases. Grey literature sources will also be searched. Two reviewers will independently screen the literature and extract data using an iteratively refined charting tool focusing on technological forms, content components, and outcomes. Findings will be synthesized through descriptive mapping and qualitative content analysis.

**Discussion:**

To our knowledge, based on preliminary searches, this appears to be the first scoping review to focus specifically on virtual community-based interventions targeted at dementia family caregivers. Findings will provide a comprehensive overview of technological forms, content components, and outcomes, offering essential insights for the design, implementation, and evaluation of future virtual community interventions.

## Introduction

Dementia poses a critical global public health challenge, with rising prevalence contributing to increased societal and caregiver burden [1]. The extensive care required by individuals living with dementia is predominantly provided by family caregivers, who deliver sustained support across daily care, emotional companionship, and health management [2–4]. As of 2023, more than 11 million unpaid caregivers in the United States alone provided an estimated 18.4 billion hours of care for people with Alzheimer’s disease or related dementia [5]. This considerable commitment is frequently accompanied by significant personal consequences, including high rates of psychological distress, sleep disturbances, social isolation, and chronic disease risk among caregivers [6–10]. Caregivers also report unmet needs for dementia-related knowledge, coping strategies, and accessible support services [11,12].

Traditional caregiver-support interventions, such as in-person training, community workshops, or clinic-based programs, face significant limitations. These barriers, including geographic dispersion, limited healthcare resources, time constraints, and workforce shortages, impede many caregivers, particularly in rural or underserved regions—from accessing sustainable support [13,14]. These practical and structural limitations have driven a growing interest in flexible, accessible, and scalable digital solutions.

Among these, virtual communities (VCs), first conceptualized by Rheingold [15] as social aggregations formed through sustained online interactions, are digital spaces where people communicate and interact through cyberspace. VCs encompass a wide range of digital environments, including social media groups, online forums, chat rooms, and mobile app-based communities [16]. In contemporary healthcare contexts, VCs are online platforms that facilitate interaction, information exchange, and emotional support, emerging as promising tools for supporting family caregivers [17,18]. By allowing for the flexible, asynchronous exchange of experiential knowledge and peer support across geographical boundaries [17,18], VCs effectively overcome structural barriers inherent in traditional support modalities and have been leveraged in contexts such as chronic disease management, mental health, cancer survivorship and professional networking [16,18–20].

Although digital interventions for dementia caregivers, such as telehealth [21], eHealth [22], and mobile apps [23], have been systematically reviewed, existing literature rarely isolates the virtual community, defined by its core feature of interactive support, as a distinct intervention modality. Additionally, while a recent scoping review focused on Virtual Community of Practice (vCoP) for collaborative learning in dementia care, it represents only one specialized form of VCs, primarily oriented toward structured knowledge-sharing and professional development [24]. In contrast, the broader concept of VCs encompasses a wider spectrum of interactions, including emotional support, peer networking and informational exchange, extending beyond the vCoP framework [25].Therefore, a broader mapping of VCs is necessary.

Evidence regarding the application of virtual communities (VCs) for dementia family caregivers remains highly fragmented. The existing literature is marked by several critical limitations, with current evidence dispersed across three key dimensions: the technological forms employed (e.g., websites, mobile apps, social media) lack systematic classification; their content components (e.g., emotional support, information sharing) are inconsistently described; and limited understanding of the full range of caregiver outcomes associated with VC-based intervention (beyond simple well-being measures). Given the rapidly expanding digital landscape and the need for scalable caregiver support, a scoping review is warranted to systematically map the breadth, characteristics, and evidence base of VC-based interventions for dementia family caregivers.

Accordingly, the primary purpose of this scoping review is to isolate virtual communities from the broader digital-health literature and synthesize the available evidence to inform future intervention design and primary research. Specifically, this review aims to map the existing evidence on virtual community-based interventions for family caregivers with people of dementia: 1. the technological forms and key platform features; 2. the content components; and 3. the caregiver-reated outcomes reported across studies.

## Methodology

This review protocol has been registered with the Open Science Framework (OSF) database (10.17605/OSF.IO/6ZDVC). The scoping review will be conducted in accordance with the Joanna Briggs Institute (JBI) methodology for scoping reviews [26]. This protocol is reported in line with the Preferred Reporting Items for Systematic Reviews and Meta-Analyses Protocols (PRISMA-P) 2015 statement (S1 File) [27], adapted where appropriate for scoping reviews. The completed scoping review will be reported according to the Preferred Reporting Items for Systematic Reviews and Meta-Analyses extension for Scoping Reviews (PRISMA-ScR) (S2 File) [28].

### Eligibility criteria

The inclusion and exclusion criteria for this scoping review are presented in Table 1.

**Table 1:** Eligibility criteria for inclusion of studies in the scoping review.

Domain	Inclusion criteria	Exclusion criteria
Population	Family or informal caregivers (spouses, children, relatives, friends, or neighbors) providing unpaid care to individuals diagnosed with dementia (including Alzheimer’s disease, vascular, or mixed dementia). No restrictions on caregiver age, gender, or region.	Caregivers of non-dementia populations or professional/paid caregivers.
Concept	Interventions delivered in a persistent, shared online environment enabling repeated multi-user interaction among caregivers and/or moderators via posting, replying, commenting, or discussion functions. Must meet three criteria: (1) shared digital platform; (2) sustained bidirectional interaction; (3) collective participation. Multi-component interventions included only if community component is clearly identifiable.	Static websites, one-way information delivery, one-to-one teleconsultation, stand-alone apps without interactive community functions
Context	Studies conducted in any country or setting	No identifiable virtual community or online interactive component.
Sources	Peer-reviewed empirical studies published in English or Chinese, including quantitative, qualitative, and mixed-methods designs. Grey literature reporting empirical findings, including doctoral dissertations, master’s theses, conference papers, and research reports, will also be considered.	Editorials, commentaries, reviews, protocols, or grey literature that do not present empirical results.

#### Participants.

Family caregivers who are currently providing care support to individuals with medically diagnosed dementia providing unpaid care, and participants can be of any age, gender, or ethnicity. Inclusion criteria includes all forms and severities of dementia, such as Alzheimer’s disease and vascular dementia. Studies involving caregivers of non-dementia populations or professional/paid caregivers will be excluded.

#### Concept.

For this review, a virtual community-based intervention is defined as an intervention delivered within a persistent, shared online environment that enables repeated multi-user interaction among caregivers and/or moderators through functions such as posting, replying, commenting, or group discussion. Eligible interventions must meet three criteria: delivered through a shared digital platform, enable sustained bidirectional interaction rather than one-way information delivery, and allow collective participation. Individual email exchanges are excluded, while email discussion lists will qualify if they meet the three criteria for a virtual community (shared platform, sustained bidirectional interaction, collective participation). Multi-component interventions will be included only if the virtual community component is described in sufficient detail to be distinguished from other elements (e.g., individual tele-education or one-to-one counselling), and if outcomes specific to the community interaction can be isolated or the community component is the predominant feature. Individualized access refers to individual entry into a wider shared community platform, not one-to-one personalized intervention.

#### Context.

Focus on various types of virtual community environments such as websites, apps, chat rooms, forums, etc. Those not based on internet or ICT will be excluded. The study is not limited to a specific country/region and broadly explores the impact of different virtual community interventions on family caregivers of people with dementia on a global scale.

#### Sources of evidence.

Peer-reviewed empirical studies published in English or Chinese will be included, including quantitative, qualitative, and mixed-methods designs, with no date restrictions applied up to the final search date. In addition, grey literature reporting empirical findings will be considered, including doctoral dissertations, master‘s theses, conference papers, and research reports. Editorials, commentaries, reviews, protocols, and grey literature that do not present empirical results will be excluded.

### Search strategy

This scoping review will use a three-step search strategy following Joanna Briggs Institute guidance [26].

#### Step 1: Initial limited search.

An initial limited search is conducted in PubMed and CINAHL to identify preliminary articles on the topic, The titles, abstracts, and index terms (MeSH/CINAHL Headings) of these articles will be analyzed to refine the key search terms, synonyms, and index words. This analysis will guide the development of a comprehensive and customized search strategy for each subsequent database.

#### Step 2: Comprehensive systematic search.

Using the finalized key search terms and corresponding database-specific index terms, a comprehensive search will be executed across all eight included databases: CINAHL, PubMed, Web of Science, Cochrane Library, Embase, PsycINFO, China National Knowledge Infrastructure (CNKI), and Wanfang Database. Block searches combining controlled vocabulary and free-text terms with Boolean operators (AND, OR) will be applied. The search strategy for PubMed is presented in Table 2 and will be adapted for each information source. No date restrictions will be applied. The search will include publications in English and Chinese. The preliminary search was conducted in January 2026; the final search was executed in March 2026. To ensure currency, the search will be rerun prior to the final analysis to identify any recently published studies.

**Table 2:** Search strategy for PubMed.

#1	Dementia	
	((“Cognitive Dysfunction”[Mesh] OR Cognitive Dysfunctions[Title/Abstract] OR Cognitive Disorder*[Title/Abstract] OR Cognitive Impairment*[Title/Abstract] OR “Dementia”[Mesh]) OR (“Alzheimer Disease”[Mesh] OR Alzheimer Syndrome[Title/Abstract] OR Alzheimer* Diseases[Title/Abstract]))	345867
#2	Family caregiver	
	(((((((“Caregivers”[Mesh]) OR (Caregiv*[Title/Abstract])) OR (Care Giver*[Title/Abstract])) OR (Carer*[Title/Abstract])) OR (Family Caregiver*[Title/Abstract])) OR (Spouse*[Title/Abstract])) OR (Informal Caregiver*[Title/Abstract])) OR (relative*[Title/Abstract])	2050615
#3	Virtual community	
	(“virtual communit*”[Title/Abstract] OR “online communit*”[Title/Abstract] OR “online support group”[Title/Abstract] OR “online support community”[Title/Abstract] OR “peer support group”[Title/Abstract] OR “peer-to-peer”[Title/Abstract] OR listserv*[Title/Abstract] OR forum*[Title/Abstract] OR “discussion board”[Title/Abstract] OR “message board”[Title/Abstract] OR “social networking site”[Title/Abstract] OR “social media group”[Title/Abstract] OR “online peer support”[Title/Abstract] OR “Facebook group”[Title/Abstract] OR “WeChat group”[Title/Abstract] OR “WhatsApp group”[Title/Abstract] OR (moderated[Title/Abstract] AND chat[Title/Abstract]) OR ((“virtual”[Title/Abstract] OR “online”[Title/Abstract] OR “digital”[Title/Abstract] OR “web-based”[Title/Abstract] OR “internet”[Title/Abstract] OR “eHealth”[Title/Abstract] OR “mobile”[Title/Abstract] OR “app”[Title/Abstract]) AND (“communit*”[Title/Abstract] OR “community of practice”[Title/Abstract] OR “forum*”[Title/Abstract] OR “group*”[Title/Abstract])))	250774
#4	#1 AND #2 AND #3	1160

#### Step 3: Supplementary searches (grey literature and citation chasing).

To ensure literature saturation and capture potentially relevant empirical evidence not published in peer-reviewed journals, supplementary searches will be performed in grey literature sources. Grey literature, including doctoral dissertations, master‘s theses, and conference papers, will be searched in ProQuest and Google Scholar. Google Scholar results will be screened by relevance order, limited to the first 10 pages, with the search date recorded. Preprint servers such as medRxiv will also be searched. When multiple records report the same study (e.g., thesis, conference abstract, preprint, journal article), the most complete peer-reviewed version will be retained; preprints will be included only if no peer-reviewed publication is available. Grey literature will be included only if it reports primary empirical findings (quantitative, qualitative, or mixed-methods) and meets the same Population-Concept-Context eligibility criteria applied to peer-reviewed studies. The reference lists of all included articles will be manually screened to identify additional relevant studies, and forward citation searches will be performed for included seminal papers.

### Study selection

Prior to formal screening, two reviewers (YW and LX) will independently screen a random sample of 20 titles/abstracts to calibrate application of eligibility criteria. Discrepancies will be discussed and eligibility criteria refined if necessary before full screening begins. After deduplication in EndNote 20, two reviewers (YW and LX) will independently screen titles/abstracts and then full texts. Reasons for exclusion will be documented. Disagreements will be resolved through discussion or consultation with a third reviewer (LZ, FZ, or HZ). Studies involving mixed samples (e.g., interventions including both dementia caregivers and other caregiver populations, or interventions targeting both caregivers and persons with dementia) will be included only if dementia caregiver-specific data can be extracted separately. Studies where the virtual community is part of a complex multi-component intervention will be included only if the community component can be clearly distinguished and described. The search results and study inclusion process will be reported in full in the final scoping review and presented in a PRISMA flowchart [29]. The JBI methodology described herein for conducting and reporting scoping reviews is consistent with the PRISMA-ScR checklist and will be followed [28].

### Data extraction

Data will be extracted from full-text papers by two reviewers (YW and LX) working independently using a JBI template-based data extraction tool [29]. The results will then be cross-checked and discussed, and the data extraction form will be continuously revised during the iterative process. Any disagreements between the two reviewers will be resolved through discussion or consultation with a third independent reviewer (XL). The authors of the study may be contacted to provide missing or additional data if necessary. If contacted, we will limit communication to two attempts over a two-week period. If key information is still unavailable after this period, the study will generally be retained and the missing information will be recorded as ‘not reported’ in the final review, unless the absence of information prevents us from answering the primary scoping review questions. The data extraction tool will be adapted as needed to allow for the iterative charting of the included evidence [30]. Extracted data will include specific details about study details, participants, virtual community characteristics, content components, comparator (if any) and results related to the review questions. For grey literature sources, the same data extraction form will be used, and the source type (e.g., dissertation, conference paper, report) will be recorded as a separate field to allow for subgroup analysis if appropriate.The specifics of the extracted data are shown in Table 3.

**Table 3:** Data extraction.

Category	Data to be extracted
Study details	Author(s), year, country, study aim, study design, theoretical framework
Participants	Sample size, caregiver relationship (spouse/adult child/mixed), dementia type/severity if reported;
Virtual community characteristics	technical form (website/ mobile app/ social media), synchronous vs asynchronous, moderator type (peer/ nurse/ psychologist/ multidisciplinary team/ none), intervention duration, frequency, intensity
Content components	Informational support, emotional support, professional support, other (specify)
Comparator (if any)	Usual care, alternative intervention, none
Outcomes	Caregiver outcomes reported (e.g., burden, depression, quality of life, self-efficacy), engagement/adherence metrics, acceptability/usability, implementation barriers/facilitators
Grey literature	Source type (dissertation/ conference paper/ report/ preprint), whether peer-reviewed version exists

### Quality assessment

Critical appraisal will not be used as an exclusion criterion in this scoping review, as the primary aim is to map the breadth of available evidence rather than to evaluate study quality for synthesis. However, to provide context for interpreting findings, methodological limitations of included studies will be descriptively characterized using the Mixed Methods Appraisal Tool (MMAT) [31]. Critical appraisal will be conducted independently by two reviewers (YW and LX), and the results will be presented in a descriptive summary alongside the mapped evidence.

### Data analysis and presentation

Data will be synthesized using: Descriptive statistics to summarize study characteristics. Qualitative content analysis to group technological forms and intervention components. Evidence mapping that visually presents relationships among technological forms, content components, and outcomes. Results will be presented through tables, narrative summaries, and thematic maps aligned with the review questions.The literature search was conducted in March 2026 and will be updated prior to the final analysis to ensure currency. As of May 2026, data extraction is underway and is expected to be completed by June 2026. Data synthesis and analysis are planned for July 2026, and the final scoping review is anticipated to be submitted for publication by August 2026.

## Discussion

To our knowledge, based on preliminary searches, this appears to be the first scoping review to focus specifically on virtual community-based interventions for family caregivers of people with dementia. This review aims to elucidate the technological forms, content components, and range of outcomes reported in existing literature, with findings anticipated to map the breadth of evidence, identify key benefits and implementation challenges, and provide recommendations for future research and intervention development.

Given the accessibility and scalability of virtual communities, mapping the existing evidence may inform the future design, implementation, and evaluation of support interventions for family caregivers of people with dementia. To comprehensively identify and map the evidence, this scoping review will be conducted according to the JBI methodology. This protocol follows PRISMA-P, and the final review will adhere to PRISMA-ScR. Literature saturation will be ensured by checking reference lists of included articles and performing forward citation searches.

## Supporting information

S1 File PRISMA-P Checklist. (DOCX)

S2 File PRISMA-ScR Checklist. (DOCX)
